# A Highly Divergent Hepacivirus Identified in Domestic Ducks Further Reveals the Genetic Diversity of Hepaciviruses

**DOI:** 10.3390/v14020371

**Published:** 2022-02-11

**Authors:** Xue-Lian Zhang, Xin-Yan Yao, Yu-Qian Zhang, Zhi-Hang Lv, Hong Liu, Jing Sun, Jian-Wei Shao

**Affiliations:** School of Life Science and Engineering, Foshan University, Foshan 528225, China; zxlsjw0312@163.com (X.-L.Z.); yaoxinyan95@163.com (X.-Y.Y.); aixxdxiaoqq@163.com (Y.-Q.Z.); lzh15637629901@163.com (Z.-H.L.); liuhong11252021@163.com (H.L.); sj1075963621@163.com (J.S.)

**Keywords:** novel hepacivirus, genetic diversity, domestic duck, China

## Abstract

Hepaciviruses represent a group of viruses that pose a significant threat to the health of humans and animals. During the last decade, new members of the genus *Hepacivirus* have been identified in various host species worldwide, indicating the widespread distribution of genetically diversified hepaciviruses among animals. By applying unbiased high-throughput sequencing, a novel hepacivirus, provisionally designated *Hepacivirus Q*, was discovered in duck liver samples collected in Guangdong province of China. Genetic analysis revealed that the complete polyprotein of *Hepacivirus Q* shares 23.9–46.6% amino acid identity with other representatives of the genus *Hepacivirus*. Considering the species demarcation criteria for hepaciviruses, *Hepacivirus Q* should be regarded as a novel hepacivirus species of the genus *Hepacivirus* within the family *Flaviviridae*. Phylogenetic analyses also indicate the large genetic distance between *Hepacivirus Q* and other known hepaciviruses. Molecular detection of this novel hepacivirus showed an overall prevalence of 15.9% in duck populations in partial areas of Guangdong province. These results expand knowledge about the genetic diversity and evolution of hepaciviruses and indicate that genetically divergent hepaciviruses are circulating in duck populations in China.

## 1. Introduction

The genus *Hepacivirus*, along with the genera *Flavivirus*, *Pegivirus*, and *Pestivirus*, currently belongs to the family *Flaviviridae*, which comprises a genetically diverse group of human and animal pathogens [1]. Members of the genus *Hepacivirus* are enveloped viruses, with unsegmented, single-stranded, positive-sense RNA genomes about 10 kb in length. The genomes of these viruses contain a single long open reading frame (ORF) encoding a single polyprotein of about 3000 amino acids (aa), which is flanked by a 5′ untranslated region (UTR) and a 3′ UTR [1]. This polyprotein is further cleaved by host and viral proteases into structural proteins (Core, E1, and E2) and nonstructural proteins (p7, NS2, NS3, NS4A, NS4B, NS5A, and NS5B) [2,3].

Hepatitis C virus (HCV), the type species of the genus *Hepacivirus*, is one of the leading causes of acute and chronic hepatitis, liver cirrhosis, and hepatocellular carcinoma in humans, and the infection rate of HCV worldwide is approximately 3%, with an estimated 58 million people suffering from chronic HCV infection (https://www.who.int/en/news-room/fact-sheets/detail/hepatitis-c; accessed on 27 July 2021). Since its first discovery in 1989, HCV has been considered as the sole representative of the genus *Hepacivirus*, and humans were regarded as the only natural host for a long time, although experimental infection of HCV in chimpanzees is possible [4]. However, since 2011, many novel genetically diversified hepaciviruses have been discovered from a wide range of mammalian hosts, such as dogs [5], horses [5], monkeys [6], bats [7], rodents [8,9], cattle [10,11], and donkeys [12]. In addition, non-mammalian hosts harboring hepaciviruses have also been described, including catshark [13], birds [14,15], fish, and other vertebrates [16,17,18]. Moreover, some highly divergent hepaciviruses were recently detected in mosquitoes and ticks, although their true host is uncertain [19,20,21]. Furthermore, a novel hepacivirus tentatively named RHV-GS2015 was recently identified in long-tailed ground squirrels in China [22]. Currently, these divergent hepaciviruses identified in different host species have been classified into 14 formal species (named *Hepacivirus A*–*N*) and some unclassified hepaciviruses, based on their phylogenetic relationships and host range [1].

Hepaciviruses in birds were first identified in domestic ducks collected over a wide geographical area of China in 2019, with the positive rate of viral RNA in the different regions ranging from 38.5–88% [14]. This infection may be associated with severe drops in egg production [14]. Since then, hepaciviruses have also been detected in the Guangdong province of China [23]. Furthermore, novel hepaciviruses were detected in Bald eagles in the USA in 2019 [15]. In the present study, a novel highly divergent hepacivirus, provisionally designated *Hepacivirus Q*, was identified in liver samples of domestic ducks collected in Guangdong province, China through an unbiased high-throughput sequencing and meta-transcriptomic analysis. We obtained the complete genome sequence and determined the genomic features, the genetic diversity, and the evolution of *Hepacivirus Q*. In addition, the prevalence of this novel hepacivirus in duck populations in partial areas of Guangdong province in China was also investigated. This study expands knowledge about the genetic diversity and evolution of hepaciviruses and shows that genetically divergent hepaciviruses are circulating in duck populations in China.

## 2. Materials and Methods

### 2.1. Sample Collection, RNA Extraction, and Meta-Transcriptome Sequencing

In April 2021, 30 liver tissues of domestic ducks were collected by authorized veterinarians of the veterinary hospital of Foshan University from a duck farm located in Zhaoqing city of Guangdong province. Approximately 50 mg of liver tissue was homogenized with 500 μL sterile phosphate-buffered saline (PBS), and total RNA was extracted from 200 μL homogenates using TRIzol LS reagent (Invitrogen, Carlsbad, CA, USA) and subsequently purified using the RNeasy Plus Mini Kit (Qiagen, Hilden, Germany). The quantity and quality of extracted RNA was evaluated with a NanoDrop 2000 (Thermo Fisher Scientific, Waltham, MA, USA). Subsequently, all RNA solutions for individual homogenates were then merged as one pool in equal quantity, and the quality of the pooled RNA was evaluated using an Agilent 2100 Bioanalyzer (Agilent Technologies, Santa Clara, CA, USA) before library construction and sequencing.

RNA library preparation was conducted following the methodology previously described [16,24]. Briefly, ribosomal RNA (rRNA) was depleted using a Ribo-Zero-Gold (Epidemiology) kit (Illumina Inc., San Diego, CA, USA), following the manufacturer’s instructions. The rest of the RNA was fragmented, reverse-transcribed, adapted, and purified using a TruSeq total RNA library preparation kit (Illumina Inc.). Library quality was examined by the Qubit (Thermo Fisher Scientific) high-sensitivity RNA/DNA assays and Agilent 2100 Bioanalyzer (Agilent Technologies). Paired-end (150 bp) sequencing was performed on the Illumina Hiseq 2500 platform. All library preparation and sequencing were performed by Novogene (Tianjin, China).

### 2.2. Bioinformatics Analyses and Genome Sequence Determination

Sequencing reads were demultiplexed, trimmed for the adaptor and quality control with the fastp program [25], and subsequently de novo assembled using the Megahit program [26] with default parameter settings. The resulting contigs were compared against the entire non-redundant protein (nr) database downloaded from GenBank using the diamond blastx program [27] with an e-value threshold of 1 × 10^−4^. These viral contigs with unassembled overlaps or from the same scaffold were merged using the SeqMan program implemented in the Lasergene software package (version 7.1, DNAstar). To confirm the assembly results, reads were mapped back to the target contigs with Bowtie2 [28], and assembly errors were inspected using the Integrated Genomics Viewer (IGV) [29]. Gaps between these contigs were filled by RT-PCR and Sanger sequencing. The genome termini of the virus were determined using 5′/3′ RACE kits (TaKaRa, Dalian, China), as described previously [30]. The final virus genome sequences were obtained for the majority consensus of the mapping assembly and confirmed by Sanger sequencing with overlapping primers that covered the entire sequence.

### 2.3. PCR Screening for This Newly Identified Hepacivirus

**Figure 1:**
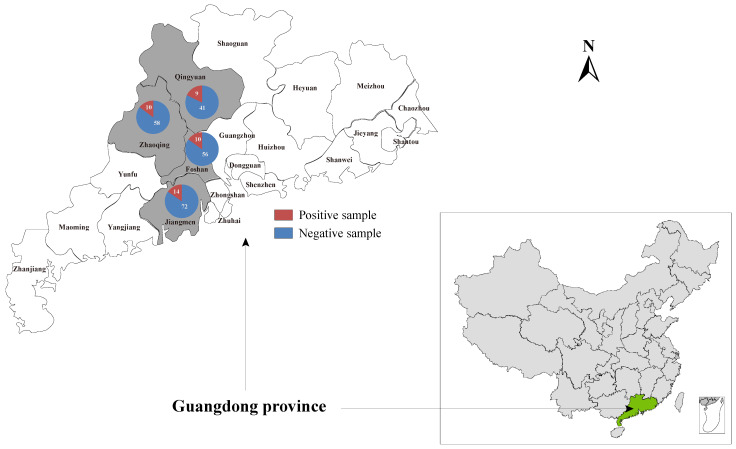
Geographic maps showing the location of sampling sites where the duck liver samples in this study were collected. This map was plotted with a combination of Surfer software version 4 (Golden Software, Golden, CO, USA) and Adobe illustrator version CC2017 (Adobe, San Jose, CA, USA).

A total of 240 liver samples were collected from domestic ducks without obvious abnormalities in Zhaoqing (*n* = 38), Qingyuan (*n* = 50), Foshan (*n* = 66), and Jiangmen (*n* = 86) cities of Guangdong province. Moreover, the 30 individual samples collected in Zhaoqing that were subjected to meta-transcriptome sequencing were also included in the investigation into the prevalence of this novel hepacivirus described herein in duck populations (Figure 1). Total RNA was prepared from 200 μL of homogenates of individual liver samples and subjected to the screening of *Hepacivirus Q* by nested PCR using the specific primer pairs targeting the NS5B coding region (Table S1). All the PCR products of the expected size were confirmed by Sanger sequencing.

### 2.4. Sequence Comparison and Phylogenetic Analyses

The prediction of potential open reading frames (ORFs) was performed by ORFfinder (https://ftp.ncbi.nlm.nih.gov/genomes/TOOLS/ORFfinder/linux-i64/; accessed on 4 May 2021) and annotated based on comparisons against the non-redundant protein database. The cleavage sites for the viral polyprotein processing were extrapolated by manually comparing the polyprotein sequence with that with the highest amino acid sequence identity, which was previously described in the Bald eagle hepacivirus (BeHV) identified in the USA [15]. *N*-glycosylation sites were predicted using NetNGlyc 1.0 (http://www.cbs.dtu.dk/services/NetNGlyc; accessed on 18 March 2012). The sequences of viruses classified to the genus *Hepacivirus* were downloaded from GenBank, and amino acid (aa) sequence identities between the sequences generated in this study along with other reference sequences were calculated by the MegAlign program available in the Lasergene software package (version 7.1, DNAstar, Madison, WI, USA).

To infer the phylogenetic relationship between the viruses identified in the present study and other known hepaciviruses, amino acid sequences of the complete polyprotein, NS3 (peptidase and helicase), and NS5 (RNA-dependent RNA polymerase) proteins were aligned using the E-INS-i algorithm implemented in the MAFFT program [31]. Phylogenetic trees were then reconstructed using the maximum likelihood method (ML) implemented in PhyML version 3.0 [32], employing the LG amino acid substitution model with a gamma (Γ)-distribution model (i.e., LG + Γ), and determined with Prot-Test 3 [33] and a Subtree Pruning and Regrafting (SPR) branch-swapping algorithm with bootstrap support values calculated from 100 replicate trees. All phylogenetic trees were mid-point rooted for purposes of clarity only.

### 2.5. Genome Recombination Analysis

The RDP, GENECONV, bootscan, maximum chi square, Chimera, SISCAN, and distance plot recombination detection methods available within RDP4 [34] were used to determine the potential recombination events that occurred in the evolutionary history of this newly identified hepacivirus. The analyses were performed based on the complete genome sequences with default settings for the different test methods and a Bonferroni corrected *p*-value cutoff of 0.05. Only sequences with significant evidence (*p* < 0.05) of recombination detected by at least two methods and confirmed by phylogenetic analysis were taken to represent strong evidence for recombination. Additionally, similarity plot analyses were inferred to further characterize potential recombination events, including the location of possible breakpoints, as implemented in Simplot version 3.5.1 [35].

## 3. Results

### 3.1. Identification of a Novel Hepacivirus in Domestic Ducks

**Figure 2:**
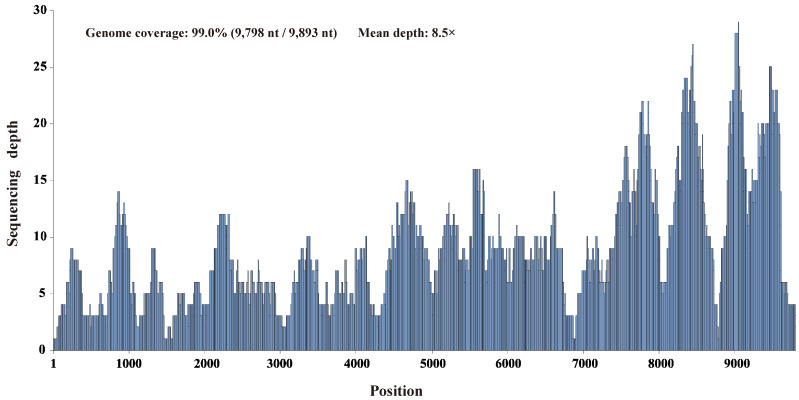
Mapped read count plot of the *Hepacivirus Q* genome. The histograms show the coverage depth per base of the *Hepacivirus Q* genome, and the mean sequencing depth was 8.5×.

Pooled RNA samples extracted from 30 individual duck liver samples were screened for known and putative novel viruses by unbiased high-throughput RNA sequencing. Through de novo assembly and comparison against the nr database using the diamond blastx program with an e-value cutoff of 1 × 10^−4^, six contigs ranging from 455 to 3016 nt in length were annotated as the Bald eagle hepacivirus of the genus *Hepacivirus* within the family *Flaviviridae*, with 37.4–65.1% amino acid identity. After filling the gaps between the contigs through RT-PCR and determining the terminal sequences using 5′/3′ RACE, this virus’s complete genome sequence was obtained. Using this sequence as the reference sequence, 563 reads were remapped to this reference sequence and provided 99.0% genome coverage (9798 nt/9893 nt) with 96.8% pairwise identity at a mean depth of 8.5× (Figure 2).

The complete genome sequence contained one ORF of 9000 nucleotides which encodes a polyprotein of 2999 amino acids (aa). The blastp search against the NCBI nr database revealed highly significant homology to the polyprotein of a hepacivirus named Bald eagle hepacivirus (BeHV). Alignments were contiguous and extended over the entire length of the polyprotein (query coverage, 100%). Results from pairwise alignments between the proteins of this newly identified virus and other known hepacivirus species are summarized in Table 1. The complete polyprotein of this novel hepacivirus shared 23.9%–46.6% amino acid identity with other representatives of the genus *Hepacivirus*. The most highly conserved regions were those encoding the NS3 and NS5B proteins, with 35.6–53.0% and 30.9–56.3% amino acid identity, respectively (Table 1). In the conserved region of NS3 (positions 1123–1566) and NS5B (amino acid positions 2536–2959), as numbered in the reference sequence of *Hepacivirus C* (NC038882), this novel virus identified herein exhibited amino acid *p*-distances of 0.463–0.600 and 0.395–0.654, respectively, with the known hepaciviruses (Table S2), which exceed the species demarcation cutoff for hepacivirus [1]. The overall pattern and degree of sequence diversity suggest that the new virus may be considered a novel species of the genus *Hepacivirus*. Following the nomenclature used for previous species, this novel virus determined in the present study was provisionally named “*Hepacivirus Q*”.

### 3.2. Genomic Characterization of Hepacivirus Q

**Figure 3:**
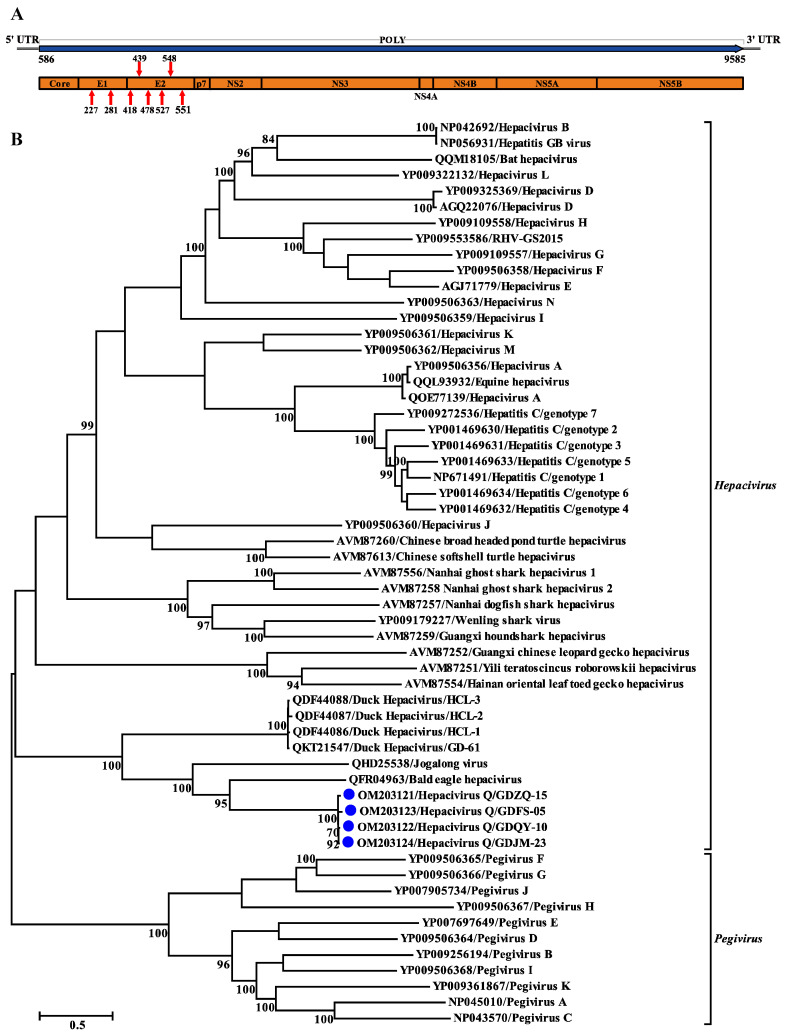
Genomic characterization and phylogenetic analysis based on the complete polyprotein of *Hepacivirus* *Q* and other reference hepaciviruses. (**A**) Genome organization of *Hepacivirus* *Q* identified in this study. Red arrows indicate N-linked glycosylation sites. (**B**) Phylogenetic analysis based on the amino acid sequence of the complete polyprotein of *Hepacivirus* *Q*. The trees were constructed based on the maximum likelihood method implemented in PhyML v3.0 and mid-point rooted for clarity; the scale bar represents the number of nucleotide substitutions per site. Bootstrap values were calculated with 100 replicates of the alignment, and only bootstrap values > 70% are shown at relevant nodes.

The genome of *Hepacivirus* *Q* consists of 9893 nucleotides and contains one large ORF encoding a polyprotein of 2999 aa. This polyprotein comprises considerably fewer amino acid residues than that of BeHV (3430 aa; MN062427) and other hepaciviruses identified in ducks (3607 aa; MK737639–MK737641 and MT135177). The putative cleavage sites specific for polyprotein processing were identified, and some of them were shown to be well conserved among the hepacivirus sequences analyzed here (Table 2). The predicted *Hepacivirus* *Q* polyprotein contained ten proteins typical for hepaciviruses in the order of Core-E1-E2-p7-NS2-NS3-NS4A-NS4B-NS5A-NS5B (Figure 3A). The large ORF is flanked by 5′ and 3′ untranslated regions (UTRs) consisting of 585 nt and 308 nt, respectively. Similar to HCV and other hepaciviruses, two and six *N*-glycosylation sites were also predicted in the putative E1 and E2 proteins of *Hepacivirus* *Q*, respectively (Figure 3A). Similar to other members of the genus *Hepacivirus*, a model Kozak sequence (AAGAUGG) at the proposed translation initiation site shows clear evidence of intrinsically disordered regions spanning the capsid protein and the 5′ half of NS5A [6]. Moreover, canonical microRNA 122 (miRNA-122) binding sites (CACUCC) were also absent in the 5′ NCR sequence of *Hepacivirus* *Q*, like other hepaciviruses identified in ducks [14].

### 3.3. Prevalence and Genetic Diversity of Hepacivirus Q in Guangdong

To gain an insight into the prevalence of *Hepacivirus* *Q* in duck populations, 270 individual duck liver samples collected from four duck farms located in Guangdong province, including those individual samples subjected to high-throughput RNA sequencing, were screened by nested RT-PCR using primers HepQ_NS5B_fwd1, HepQ_NS5B_fwd2, and HepQ_NS5B_rev (listed in Table S1). Using these primer pairs, a total of 43 positive individual samples collected from distinct duck farms were detected (Figure 1). The overall prevalence of *Hepacivirus Q* in duck populations in Guangdong province was 15.9%, while the prevalence in the four distinct duck farms ranged from 14.7% to 18.0%. Four complete genome sequences of *Hepacivirus* *Q* variants were obtained by overlapping nested RT-PCR and 5′/3′ RACE (primers listed in Table S1) from positive samples and have been submitted to the GenBank under accession numbers OM203121 to OM203124. The *Hepacivirus* *Q* sequences identified herein showed low genome sequence diversity at the nucleotide level, ranging from 0.7 to 4.8%. The pairwise amino acid distances indicated low divergence among the four *Hepacivirus* *Q* sequences from this study but high divergence from other representative hepaciviruses (Table S3).

### 3.4. Phylogenetic and Recombination Analyses of Hepacivirus Q

**Figure 4:**
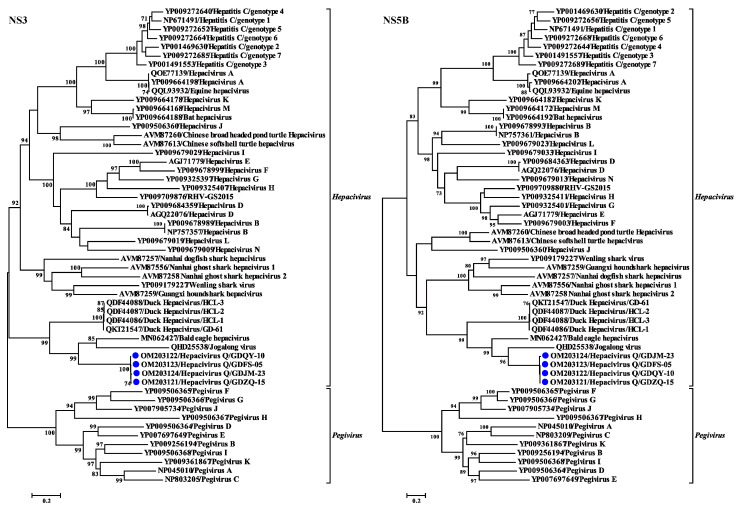
Phylogenetic analysis based on amino acid sequence of NS3 and NS5B proteins of hepaciviruses. The trees were constructed based on the maximum likelihood method implemented in PhyML v3.0 and mid-point rooted for clarity; the scale bar represents the number of nucleotide substitutions per site. Bootstrap values were calculated with 100 replicates of the alignment, and only bootstrap values >70% are shown at relevant nodes.

Phylogenetic trees reconstructed based on the amino acid sequence of the complete polyprotein, NS3, and NS5B proteins support the grouping of the newly identified virus within the genus *Hepacivirus*. All the phylogenetic trees showed a similar topology such that the four *Hepacivirus* *Q* strains formed a separate cluster distantly related to other hepaciviruses (Figure 3B and Figure 4). Moreover, the *Hepacivirus* *Q* strains branch very deeply next to DuHVs, BeHV, and the Jagolong virus (JgV) and form the potential avian clade. Additionally, *Hepacivirus* *Q* strains exhibited great genetic divergence from other duck hepaciviruses (HCL-1, -2, and -3, and GD-61), although they were also identified in ducks in China, suggesting the high genetic diversity of hepacivirus. Notably, *Hepacivirus* *Q* strains formed a sister lineage with BeHV in the phylogenetic tree estimated based on the complete polyprotein. Still, they showed a closer relationship with BeHV and JgV in the NS3 and NS5B trees, respectively (Figure 3 and Figure 4). The phylogenetic incongruence suggests that recombination events may have occurred in the evolutionary history of *Hepacivirus* *Q*, although no statistically supported recombination event was detected within *Hepacivirus* *Q* strains or other hepaciviruses after systematic analyses.

## 4. Discussion

HCV has been considered as the only member of the genus *Hepacivirus* since its discovery in 1989. The first identification of hepaciviruses in dogs opened a window for a broad host spectrum of the genus *Hepacivirus* [36], and various hepaciviruses have been discovered from a wide range of host species [5,6,7,8,9,10,11,12,13,14,16,17,18], suggesting the ubiquitous presence of this highly variable virus. Here, a thus far unknown hepacivirus was identified in ducks and was named *Hepacivirus* *Q*. Estimation of evolutionary distances and phylogenetic analyses showing that the sequences of the four *Hepacivirus* *Q* strains identified in different duck populations are closely related to each other but display large genetic distances from other previously described hepaciviruses suggest the extensive phylogenetic and host diversity of the genus *Hepacivirus*.

*HepacivirusQ* is a highly divergent hepacivirus and shares only 46.6% aa identity with its closest relative, Bald eagle hepacivirus, across the complete polyprotein. Noticeably, all the mature proteins of *Hepacivirus* *Q* display a closer relationship with BeHV, except the most conserved protein, NS5B, which exhibits a higher sequence identity with JgV than BeHV (Table 1). Moreover, the incongruence displayed in the phylogenetic trees reconstructed based on the NS3 and NS5B proteins (Figure 4) also indicates that recombination events may have occurred in the evolutionary history of *Hepacivirus* *Q*. However, no statistically supported recombination event was detected within *Hepacivirus* *Q* strains or other hepaciviruses after systematic analyses, although genetic recombination is an important evolutionary mechanism for HCV [37] and recombination events have been determined in other hepaciviruses [38,39], even in duck hepaciviruses [23]. Nevertheless, the large genetic distance from other known hepacivurses and the limitation of the data set hamper definite assertions on the presence or absence of recombination in *Hepacivirus* *Q*.

The vast majority of hepaciviruses identified to date are highly species-specific and are thought to be experiencing long-term coevolution and host adaptation [40]. In the present study, the phylogenetic placement of *Hepacivirus* *Q* with DuHVs, JgV, and BeHV indicates that they form a potential avian clade of hepacivruses, although they exhibit large genetic distances. In addition, the distant phylogenetic relationship between this potential avian clade and all other hepaciviruses indicates that hepaciviruses infecting avian hosts could be expected to diverge from mammalian, reptilian, or fish hepaciviruses, and also suggests a notably different evolutionary history for hepaciviruses in different host species.

As a human pathogen distributed worldwide, HCV poses a great threat to human health, causing liver failure, hepatitis, and hepatocellular carcinoma. Considerably diversified hepaciviruses have been identified in various animal species. However, animal hepaciviruses have been linked to clinical diseases in only a few cases [10,41]. Although duck hepacivirus was recently discovered in a study conducted to investigate the etiology of severely diseased ducks, the pathogenicity of duck hepacivirus is unclear as the virus was also highly prevalent in healthy ducks [14]. Similarly, *Hepacivirus* *Q* was also identified in liver samples randomly collected from duck populations and it cannot be associated with any clinical disease. The high prevalence of *Hepacivirus* *Q* in duck populations suggests that the virus may be non-pathogenic in ducks, and persistent infection may be established in the infected ducks. Future studies should investigate the epidemiology of *Hepacivirus* *Q* in broader areas and populations and the pathogenicity of this novel virus in ducks.

In conclusion, a thus far unknown hepacivirus was identified in duck samples collected from Guangdong province of China through unbiased RNA sequencing, which expands knowledge concerning the genetic diversity and evolution of hepacivirues and indicates that genetically divergent hepaciviruses are circulating in duck populations in China.

## Figures and Tables

**Table 1:** Amino acid sequence similarity of mature *Hepacivirus Q* proteins compared to those of other hepaciviruses. Sequences were aligned using the ClustalW method and the calculated sequence similarity implemented in MegAlign.

		Value for the Indicated Virus in Relation to *Hepacivirus Q*																	
Protein	A	B	C	D	E	F	G	H	I	J	K	L	M	N	RHV	JgV	HCL1	GD61	BeHV
Polyprotein	23.9	24.6	23.7	24.6	24.8	24.0	24.0	24.0	24.1	24.7	24.6	25.5	25.4	24.1	25.0	39.1	35.5	35.6	46.6
Core	26.5	24.1	23.9	25.2	24.7	28.1	26.5	26.5	23.9	28.6	25.8	24.8	27.5	23.1	24.1	9.4	28.8	28.2	47.6
E1	24.6	20.8	19.4	19.7	22.5	19.3	18.5	17.6	18.7	26.8	22.9	16.0	23.9	16.1	18.2	45.2	38.6	38.6	55.6
E2	11.6	13.2	15.0	15.0	15.9	16.5	17.0	15.2	12.8	9.3	12.1	15.0	12.5	13.7	14.8	29.7	30.9	31.7	44.6
p7	21.0	17.5	27.4	15.6	11.1	10.9	15.1	20.0	17.3	18.6	33.9	15.3	27.4	15.0	19.6	31.1	31.5	31.5	47.3
NS2	15.6	15.7	17.0	16.1	15.8	15.8	16.8	17.9	17.8	17.8	17.5	18.8	17.5	15.3	14.3	32.2	29.9	29.9	41.9
NS3	37.2	38.9	37.5	39.2	35.9	37.6	36.7	35.6	35.6	38.8	38.1	41.2	37.6	37.3	40.2	46.6	48.1	48.3	53.0
NS4A	17.6	25.5	21.6	26.9	23.5	25.5	19.6	17.3	21.2	15.4	21.2	27.5	25.0	23.5	29.4	42.3	28.8	28.8	46.2
NS4B	21.6	21.8	22.0	21.8	20.2	16.8	17.5	22.1	22.4	24.1	23.5	19.4	24.8	23.2	19.3	35.0	32.4	32.4	41.6
NS5A	12.0	11.9	10.4	11.7	15.7	11.6	10.8	12.3	12.3	13.6	11.4	9.2	12.1	15.5	9.8	19.7	12.1	11.7	27.2
NS5B	31.6	31.6	31.1	34.0	31.4	30.9	32.1	32.1	33.0	34.4	34.2	34.6	35.1	32.0	32.2	56.3	43.1	43.3	54.8

A to N indicate *Hepacivirus A* to *Hepacivirus N*, and RHV, JgV, HCL1, GD61, and BeHV indicate RHV-GS2015, Jogalong virus, DuHV-HCL1, DuHV-GD61, and Bald eagle hepacivirus, respectively. GenBank accession numbers for the sequences are as follows: *Hepacivirus A*, NC038425; *Hepacivirus B*, NC001655; *Hepacivirus C***,** NC038882; *Hepacivirus D*, NC031950; *Hepacivirus E*, KC815310; *Hepacivirus F*, NC038427; *Hepacivirus G*, NC025672; *Hepacivirus H*, NC025673; *Hepacivirus I*, NC038428; *Hepacivirus J*, NC038429; *Hepacivirus K*, NC038430; *Hepacivirus L*, NC031916; *Hepacivirus M*, NC038431; *Hepacivirus N*, NC038432; RHV-GS2015, NC040815; Jogalong virus, MN133813; Bald eagle hepacivirus, MN062427; DuHV-HCL1, MK737640; DuHV-GD61, MT135177.

**Table 2:** Comparison of predicted cleavage sites of hepacivirus polyprotein.

	Cleavage Site at:								
Virus	C/E1	E1/E2	E2/p7	P7/NS2	NS2/NS3	NS3/NS4A	NS4A/NS4B	NS4B/NS5A	NS5A/NS5B
*Hepacivirus A*	GEA/SVV	VSC/DNY	AEA/YLS	AWA/FDN	RLL/SPI	TQT/NAW	EEC/FDH	QNC/DFT	ESC/SLS
*Hepacivirus B*	CSG/ARV	IEA/TSG	MAA/GLP	ASA/FDT	AIT/APF	VNT/SGT	EEC/ASF	DDC/GLI	FSC/SMS
*Hepacivirus C*	ASA/YQV	VDA/ETH	AEA/ALE	AYA/LDT	RLL/API	VVT/STW	EEC/SQH	TPC/SGS	VCC/SMS
*Hepacivirus D*	GAS/CVV	VTS/TSL	AAA/AAM	AVG/FDD	MLN/PFS	NDC/SLV	EEC/SFG	AQC/DGG	AKC/ASW
*Hepacivirus E*	ATA/VSN	AAA/AAP	AYA/FTP	AYA/ISL	KYT/IPF	FFA/SGY	EEC/YNW	DLC/TPP	HSC/SMS
*Hepacivirus F*	GGA/VTN	VKA/LAL	AFA/FTP	SAY/SLN	ERT/APF	YFA/STT	EEC/YQW	EDC/SCR	HEC/SSW
*Hepacivirus G*	ASA/GIF	VAA/PVS	VGA/LEV	EAY/EGG	RFT/APF	YFA/ETV	EEC/STQ	DVC/TSP	TDC/SWS
*Hepacivirus H*	AEA/NLL	SAV/AVP	SEA/VPT	RAE/QFD	QLT/KPF	YYC/GLV	EEC/ANE	EIC/DGS	SSC/SKS
*Hepacivirus I*	VEP/KPL	SVA/APV	YAQ/PPL	VEA/FSS	QLS/SPV	ELA/STW	EEC/ALD	EPC/TDS	ETC/TYS
*Hepacivirus J*	AVS/HWC	AEG/LPF	ANA/LVL	AQG/GCL	RLT/APF	EEM/TDG	EEC/GFD	AEC/AGG	TSC/NYS
*Hepacivirus K*	GEA/SYA	AQA/NPI	ADA/ALT	AVG/GPY	RHC/SPI	DDT/STG	EEC/LSY	SEC/AFF	DEC/SAS
*Hepacivirus L*	AES/VPA	AAA/MPV	AWG/WPA	AQA/ASL	ERN/APM	YSA/GGL	EEC/MQT	AEC/DGM	SKM/SRS
*Hepacivirus M*	VDA/SFA	SQA/AEH	AEG/AME	VGG/GPV	RHC/SPI	TPT/SAW	EEC/ADY	RNC/SCS	SPC/SAS
*Hepacivirus N*	VSG/YRQ	VEA/TTT	ATA/ALL	VTA/LDF	APC/SPI	LDV/WGA	EEC/WGF	VPC/GFN	KEC/SYS
JgV	AVA/FSD	AQA/GTH	IEG/AVN	VAG/FWF	KLA/API	SAG/LTV	EEC/AST	TNC/TSP	VCC/GES
DuHV-HCL1	ASA/DHI	GMA/DRS	AEG/MLS	VLG/ASV	QYT/API	NCS/AAY	EEC/SAE	YEC/NSE	ESC/SFS
DuHV-GD61	ASA/DHI	GMA/DRS	AEG/MLS	VLG/ASV	QYT/API	NCS/AAY	EEC/SAE	YEC/NSE	ESC/SFS
BeHV	ADS/SHD	VEG/GLQ	VTA/AVN	VSG/TEV	NWS/APL	VSC/SLY	EEC/AGN	YDC/ANS	HSC/SAS
Hepacivirus Q	EPA/THL	VEG/GLV	VGA/AIN	VAG/SEI	RWS/APF	VNC/SML	EEC/SSS	QLC/SSN	CSC/SMS

Cleavage is indicated by a slash (/). The GenBank accession numbers for the corresponding sequences are same as shown in Table 1.

## Data Availability

The sequences generated in this study have been submitted to GenBank under accession numbers OM203121 to OM203124.

## Supplementary Materials

The following are available online at https://www.mdpi.com/article/10.3390/v14020371/s1, Table S1: Primers used in this study, Table S2: Genetic divergence of *Hepacivirus Q* compared to other hepaciviruses indicated by the amino acid *p*-distances in the conserved region of NS3 (positions 1123–1566) and NS5B (amino acid positions 2536–2959) as numbered in the reference sequence of *Hepacivirus C* (NC038882), Table S3: Pairwise amino acid distances among *Hepacivirus Q* identified in this study and other hepaciviruses.
