# Supplementary material for: A Highly Divergent Hepacivirus Identified in Domestic Ducks Further Reveals the Genetic Diversity of Hepaciviruses
Source: Viruses. 2022 Feb 11;14(2):371. doi: 10.3390/v14020371 (PMC8879383; doi:10.3390/v14020371)
Supplement: Supplementary file 1 [file viruses-14-00371-s001.zip › Table S1.pdf]

Table S1. Primers used in this study.

Primers	Sequences (5′→3′)	Tm (°C)	Amplicon (bp)	Usage
Gap12-F	GTTAGAGGAGCCGAGGATGTC	53	1000	Fill gaps
Gap12-R1	GCAAGCCAGTCCAAACGATAG	53		between
Gap12-R2	GGTCCAAACTGAGCCATTGC	53		contigs
Gap23-F1	GCTGGTAGGAATGTTAGGTTGA	53	400	
Gap23-F2	ACTGAGAATCTAACTGGCAGGA	53		
Gap23-R	CCACAAGCAAGAGAATGAGAAG	53		
Gap34-F1	CCGACAATGGGTTCCTGAATG	52	500	
Gap34-F2	CCCGAAAGGTCACGCAATG	52		
Gap34-R	GAGCACGAGACAAGACTAAGC	52		
Gap45-F	GGCTCGAAGGTGGTAATGTG	53	2400	
Gap45-R1	ACTCGTCAAAGCAACTGGAAAT	53		
Gap45-R2	GGTAATGTGCAACTGGATGGTA	53		
Gap56-F	ACACAACCTCTAGCAACAACTG	53	400	
Gap56-R1	GACCTACAGCAAGCCACCTA	53		
Gap56-R2	GCGAACATGAGCAGATTACCA	53		
5′GSP-R1	GCACTGAGGCAACGACTCGCTTACC	63	700	Terminal
5′GSP-R2	TGGTGCTGTTGAAGTAACTGAC	55		sequence
3GSP-F1	GCTAGGTGGCTTGCTGTAGGTCTCATTG	63	500	amplification
3′GSP-F2	GGCTTGCTGTAGGTCTCATTG	55		
HepQ_NS5B_fwd1	GCGTTACATCTGCTATCCTCCT	53	835	Positive
HepQ_NS5B_fwd2	AAGATGGTCCTCGGTGATGTT	53		sample
HepQ_NS5B_rev	CGAAGGTGAGTTGAATGGTGTT	53		screening
HepQ-236F	CGGCTACGGCAGTTACAGT	55	1665	Complete
HepQ-321F	GTCTACGCTCGTGCTTCCT	55		genome
HepQ-1985R	GAGGCAACAACTCGCTTACC	55		sequence
HepQ-1658F	ACTGTGTTGAAGGTGGATTAGT	53	1671	amplification
HepQ-1682F	ACCGTAGTATGCCTAGAGTCAA	54		from positive
HepQ-3352R	CACCACAAGCAAGAGAATGAGA	54		sample
HepQ-3327F	TGCTTCTCATTCTCTTGCTTGT	54	1523	
HepQ-4911R	TTCGTCCACAGTACCATCATTC	54		
HepQ-4852R	CACCTCGACCAGTTCTACCT	54		
HepQ-4689F	ATCCACTGGCTATAATGGCAAT	53	1727	
HepQ-4760F	TTGACTTGGCTCCAACTGTTAG	54		
HepQ-6487R	AGCGGTAACGGTTCAGACA	54		
HepQ-6146F	TGCTTAATCTGCTTCACTCACT	53	1692	
HepQ-7967R	GTTAGGTAAACACGGTCTCTCC	54		
HepQ-7837R	CTTGGTCATTGGATGGGTCAT	53		
HepQ-7638F	GAGAGCAACTTCCTTGACTGAG	54	1423	
HepQ-7702F	CCGACTTCTAGTACCGAGGAG	54		
HepQ-9124R	GACCTACAGCAAGCCACCTA	54		
HepQ-8900F	CCATTCTCCCTGTTTACTCCCT	55	877	
HepQ-8908F	CCTGTTTACTCCCTGGACTTGA	55		
HepQ-9784R	AAGCAACTGGCGTCACTCA	55		
