# Supplementary material for: A Highly Divergent Hepacivirus Identified in Domestic Ducks Further Reveals the Genetic Diversity of Hepaciviruses
Source: Viruses. 2022 Feb 11;14(2):371. doi: 10.3390/v14020371 (PMC8879383; doi:10.3390/v14020371)
Supplement: Supplementary file 1 [file viruses-14-00371-s001.zip › Table S2.pdf]

Table S2. Genetic divergence of *Hepacivirus Q* compared to other hepaciviruses indicated by the amino acid *p*-distances in the conserved region of NS3 (positions 1123–1566) and NS5B (amino acid positions 2536–2959) as numbered in the reference sequence of *Hepacivirus C* (NC038882).

	Value of *p*-distance for the indicated virus in relation to Hepacivirus Q																		
Protein	A	B	C	D	E	F	G	H	I	J	K	L	M	N	RHV	JgV	HCL1	GD61	BeHV
NS3 (1123–1566)	0.589	0.579	0.570	0.573	0.613	0.586	0.600	0.600	0.600	0.582	0.568	0.543	0.573	0.578	0.563	0.527	0.513	0.510	0.463
NS5B (2536–2959)	0.654	0.649	0.649	0.618	0.654	0.650	0.644	0.650	0.628	0.606	0.636	0.616	0.611	0.651	0.640	0.397	0.537	0.535	0.395

A to N indicated the *Hepacivirus A* to *Hepacivirus N*, and RHV, JgV, HCL1, GD61 and BeHV indicated RHV-GS2015, Jogalong virus, DuHV-HCL1, DuHV-GD61, and Bald eagle hepacivirus, respectively. GenBank accession numbers for the sequences are as follows: *Hepacivirus A*, NC038425; *Hepacivirus B*, NC001655; *Hepacivirus C*, NC038882; *Hepacivirus D*, NC031950; *Hepacivirus E*, KC815310; *Hepacivirus F*, NC038427; *Hepacivirus G*, NC025672; *Hepacivirus H*, NC025673; *Hepacivirus I*, NC038428; *Hepacivirus J*, NC038429; *Hepacivirus K*, NC038430; *Hepacivirus L*, NC031916; *Hepacivirus M*, NC038431; *Hepacivirus N*, NC038432; RHV, NC040815; Jogalong virus, MN133813; Bald eagle hepacivirus, MN062427; DuHV-HCL1, MK737640; DuHV-GD61, MT135177.
