# Supplementary material for: A Highly Divergent Hepacivirus Identified in Domestic Ducks Further Reveals the Genetic Diversity of Hepaciviruses
Source: Viruses. 2022 Feb 11;14(2):371. doi: 10.3390/v14020371 (PMC8879383; doi:10.3390/v14020371)
Supplement: Supplementary file 1 [file viruses-14-00371-s001.zip › Table S3.pdf]

Table S3. Pairwise amino acid distances among *Hepacivirus Q* identified in this study and other hepaciviruses.

**Strain**	**1**	**2**	**3**	**4**	**5**	**6**	**7**	**8**	**9**	**10**	**11**	**12**	**13**	**14**	**15**	**16**	**17**	**18**	**19**	**20**	**21**	**22**	**23**
**1 *Hepacivirus A***																							
**2 *Hepacivirus B***	0.720																						
**3 *Hepacivirus C***	0.510	0.722																					
**4 *Hepacivirus D***	0.722	0.636	0.732																				
**5 *Hepacivirus E***	0.721	0.636	0.724	0.640																			
**6 *Hepacivirus F***	0.724	0.642	0.733	0.651	0.356																		
**7 *Hepacivirus G***	0.722	0.637	0.737	0.675	0.465	0.491																	
**8 *Hepacivirus H***	0.726	0.653	0.728	0.659	0.542	0.549	0.549																
**9 *Hepacivirus I***	0.734	0.668	0.731	0.663	0.660	0.666	0.661	0.663															
**10 *Hepacivirus J***	0.722	0.705	0.725	0.725	0.717	0.720	0.719	0.726	0.718														
**11 *Hepacivirus K***	0.636	0.716	0.641	0.718	0.716	0.727	0.725	0.716	0.711	0.720													
**12 *Hepacivirus L***	0.706	0.597	0.703	0.613	0.627	0.635	0.633	0.645	0.646	0.706	0.711												
**13 *Hepacivirus M***	0.625	0.712	0.645	0.719	0.706	0.718	0.716	0.717	0.706	0.713	0.503	0.710											
**14 *Hepacivirus N***	0.725	0.654	0.729	0.666	0.670	0.676	0.679	0.677	0.691	0.712	0.722	0.648	0.726										
**15 RHV-GS2015**	0.716	0.630	0.718	0.654	0.487	0.490	0.504	0.534	0.645	0.714	0.714	0.609	0.706	0.671									
**16 Jogalong virus**	0.786	0.767	0.785	0.782	0.771	0.779	0.769	0.774	0.763	0.771	0.785	0.755	0.774	0.768	0.770								
**17 DuHV-HCL1**	0.769	0.761	0.769	0.769	0.759	0.758	0.764	0.758	0.751	0.744	0.760	0.737	0.748	0.755	0.743	0.675							
**18 DuHV-GD61**	0.769	0.761	0.770	0.770	0.759	0.758	0.763	0.759	0.752	0.743	0.759	0.738	0.748	0.755	0.743	0.676	0.027						
**19 Bald eagle hepacivirus**	0.778	0.765	0.778	0.782	0.766	0.772	0.765	0.771	0.765	0.763	0.771	0.762	0.764	0.767	0.762	0.597	0.689	0.689					
**20 *Hepacivirus Q*/GDZQ-15**	0.772	0.753	0.771	0.760	0.762	0.770	0.768	0.766	0.767	0.757	0.768	0.752	0.755	0.760	0.758	0.613	0.651	0.652	0.536				
**21 *Hepacivirus Q*/GDQY-10**	0.772	0.752	0.770	0.760	0.761	0.770	0.767	0.766	0.767	0.757	0.767	0.752	0.755	0.760	0.758	0.610	0.648	0.649	0.534	0.017			
**22 *Hepacivirus Q*/GDFS-05**	0.770	0.753	0.770	0.759	0.761	0.769	0.768	0.765	0.767	0.757	0.767	0.752	0.755	0.760	0.759	0.610	0.649	0.650	0.534	0.016	0.006		
**23 *Hepacivirus Q*/GDJM-23**	0.769	0.754	0.772	0.762	0.761	0.769	0.770	0.767	0.767	0.756	0.767	0.753	0.754	0.760	0.759	0.609	0.652	0.653	0.536	0.031	0.025	0.023	

GenBank accession numbers for the sequences are as follows: *Hepacivirus A*, NC038425; *Hepacivirus B*, NC001655; *Hepacivirus C*, NC038882; *Hepacivirus D*, NC031950; *Hepacivirus E*, KC815310; *Hepacivirus F*, NC038427; *Hepacivirus G*, NC025672; *Hepacivirus H*, NC025673; *Hepacivirus I*, NC038428; *Hepacivirus J*, NC038429; *Hepacivirus K*, NC038430; *Hepacivirus L*, NC031916; *Hepacivirus M*, NC038431; *Hepacivirus N*, NC038432; RHV-GS2015, NC040815; Jogalong virus, MN133813; Bald eagle hepacivirus, MN062427; DuHV-HCL1, MK737640; DuHV-GD61, MT135177.
